# Association of CKD-MBD Markers with All-Cause Mortality in Prevalent Hemodialysis Patients: A Cohort Study in Beijing

**DOI:** 10.1371/journal.pone.0168537

**Published:** 2017-01-03

**Authors:** Duo Li, Ling Zhang, Li Zuo, Cheng Gang Jin, Wen Ge Li, Jin-Bor Chen

**Affiliations:** 1 Department of Nephrology, First Affiliated Hospital of Wenzhou Medical University, Wenzhou, China; 2 Department of Nephrology, China-Japan Friendship Hospital, Beijing, China; 3 Department of Nephrology, People’s hospital, Peking University, Beijing, China; 4 School of Social Development and Public Policy, Beijing Normal University; 5 Division of Nephrology, Department of Internal Medicine, Kaohsiung Chang Gung Memorial Hospital and Chang Gung University College of Medicine, Kaohsiung, Taiwan; Universidade de Sao Paulo, BRAZIL

## Abstract

The relationships between all-cause mortality and serum intact parathyroid hormone (iPTH), calcium, and phosphate are fairly diverse in patients on maintenance hemodialysis according to prior studies. This study evaluated the association of chronic kidney disease-mineral and bone disorder (CKD-MBD) markers with all-cause mortality in prevalent hemodialysis patients from 2007 to 2012 in Beijing, China. A cohort, involving 8530 prevalent hemodialysis patients who had undergone a 6–70 months follow-up program (with median as 40 months) was formed. Related data was recorded from the database in 120 hemodialysis centers of Beijing Health Bureau (2007 to 2012). Information regarding baseline demographics, blood CKD-MBD markers and all-cause mortality was retrospectively reviewed. By using multivariate Cox regression model analysis, patients with a low iPTH level at baseline were found to have greater risk of mortality (<75pg/ml, HR = 1.36, 95% confidence interval (CI) 1.16–1.60) than those with a baseline iPTH level within 150–300 pg/ml. Similarly, death risk showed an increase when the baseline serum calcium presented a low level (<2.1mmol/L, HR = 1.54; 95% CI 1.37–1.74). Levels of baseline serum phosphorus were not associated with the risk of death. Similar results appeared through the baseline competing risks regression analysis. Patients with a lower level of serum iPTH or calcium are at a higher risk of all-cause mortality compared with those within the range recommended by Kidney Disease Outcome Quality Initiative (KDOQI) guidelines.

## Introduction

It is common that the abnormalities in serum calcium (Ca), phosphorus (P), and parathyroid hormone (PTH) levels appear in patients with moderate and advanced chronic kidney diseases (CKD). CKD-mineral and bone disorder (CKD-MBD), manifested by the above abnormal laboratory markers, and other abnormalities such as vitamin D metabolism and bone turnover etc, contribute to the increased morbidity and mortality in patients on maintenance dialysis. Results from several studies, mainly from the United States, indicated that an increased mortality risk was associated with high intact parathyroid hormone (iPTH), Ca, and/or P in maintenance hemodialysis patients [1, 2]. Another retrospective cohort study from Canada showed that the appearance of both serum P level greater than 6 mg/dl and serum Ca level greater than 11 mg/dl was associated with a heightened risk of mortality when these two parameters were analyzed as either time-dependent or cumulative time-dependent variables in hemodialysis patients. In this study, however, no relationship was found between the iPTH levels and all-cause mortality or other outcomes [3]. A large cohort of 16,173 hemodialysis patients from six Latin American countries, the CORES Study, found that both elevated and reduced serum levels of albumin-corrected Ca, P and iPTH were associated with increments in either all-cause or cardiovascular mortalities [4]. Data from an observational study in European hemodialysis population demonstrated that patients with iPTH, Ca and P levels within the Kidney Disease Outcomes Quality Initiative (KDOQI) target ranges had the lowest risk of mortality [5], which was similar to the results of CORES Study. Recently, a prospective study from Japan indicated that both serum Ca and P levels were important predictors of the absolute risk of death in hemodialysis patients with secondary hyperparathyroidism [6].

There are lack of data from the Chinese population in order to identify the relationship between mortality and CKD-MBD markers in hemodialysis patients. Herein, we presented the analysis result from a large historical cohort study among the prevalent hemodialysis patients in Beijing, over five-year observation.

## Materials and Methods

### Study population

Beijing Hemodialysis Quality Control and Improvement Center (BJHDQCIC) was set up by the Beijing Health Bureau in 2003. One of the major missions of BJHDQCIC was to do end-stage renal disease (ESRD) registration [7, 8]. From 2007, BJHDQCIC started to collect patient-level data from 120 HD facilities in Beijing and an annual data report (ADR) has been issued thereafter. Up to 2012 June 30th there was a report of 12,144 maintenance hemodialysis patients from 120 hemodialysis facilities in Beijing by BJHDQCIC. 8,530 cases had complete records that allowed to identify categories demography, primary cause of ESRD, serum Ca, serum P and iPTH levels. Duration of the follow-up was 6~70 months (median, 40 months). Exclusion criteria: patients under 18 years old, less than 90 days on hemodialysis, incomplete data on key parameters (serum Ca, P, iPTH).

### Study design

This retrospective cohort study was targeted on the relationship between CKD-MBD markers and all-cause mortality. Baseline data from the registration system were collected, including demographics and blood CKD-MBD markers. Measurements in blood samples were performed using commercial kits and auto-analyzer equipment. Ca levels were calculated, using the following equation: measured total Ca (mg/dL) + 0.8 [4.0 –serum albumin (g/dL)] while iPTH was measured by commercial kits. A standard assay was not used in all facilities. But second-generation assays were used for iPTH measurements, either by radioimmunoassay or by electrochemiluminescence immunoassay.

In the present study, we adopted the target ranges recommended by the KDOQI guidelines as reference for analysis. Combined KDOQI and KDIGO guidelines were used to set groups and the cutoff values.

Information from all patients was anonymized and de-identified prior to analysis. The protocol for this study was approved by the Committee on Human Research at China-Japan Friendship Hospital, and the study was conducted in accordance with the Declaration of Helsinki.

### Statistical analysis

For statistical description, variables which followed a normal distribution were described by mean and standard deviation, with median otherwise described.

We used multivariate Cox regression analysis to evaluate the hazard ratios (HRs) for mortality associated with all parameters. Competing risk model was also applied to estimate the risk factors for the all-cause mortality in maintenance hemodialysis patients. The association of iPTH level as a continuous variable with mortality was also examined by Cox regression model. For this study, patients that died of all-cause, were considered as ‘events’ of interest. Patient who gave up treatment was also considered as an ‘event’. However, patients who were transferred to peritoneal dialysis or who underwent kidney transplant were termed as ‘competing risk events’. Survivals at the end of the study were described as ‘censored’.

All statistical analyses were performed by using the Stata Software (version 12.0, STATA Corporation, USA.). A P-value of <0.05 was recognized as statistically significant.

## Results

Among all the 8,530 patients, 1,243 (14.6%) died during the study period, including 113 (1.3%) of them who gave up hemodialysis treatment. 243 patients (2.8%) either underwent kidney transplant or were transferred to peritoneal dialysis. A total of 6,931 (81.3%) patients were alive at the end of the study.

### Baseline characteristics of the enrolled patients

The baseline data of these patients is described on Table 1. The average age of patients was 55.3±15.0 years old (yrs). 3,062 patients (35.9%) were under 50 yrs and 3,821 patients (44.8%) were between 50 and 70 years of age. 1,647 patients (19.3%) were at 70 years of age or older. Of all the patients, 4,539 were males (53.0%) and 3,991(47.0%) were females. The primary cause of ESRD in all the prevalent hemodialysis patients from Beijing was glomerulonephritis (n = 3,367, 39.5%), followed by diabetic nephropathy (n = 2,534, 29.4%).

**Table 1:** Baseline characteristics of patients involved in this study.

Variable	Mean	SD	N
Age(years)	55.3	15.021	8530
Gender(Female = 0, Male = 1)	0.53*	0.499	8530
iPTH(pg/ml)	351.6	403.808	8530
Serum calcium(mmol/L)	1.8	0.695	8530
Serum phosphorus(mmol/L)	3.0	1.066	8530

* Gender is dummy variable, with its mean indicating the male account for 53%, It is a Bernoulli random variable, and Standard Deviation is 0.499. The formula for SD of binary variable is square root of n*p(1-p).

Considering the baseline iPTH category, the characteristics of patients are listed on Table 2. 26.4% of the patients were within the optimal level of iPTH recommended by K/DOQI(150~300pg/ml). Low levels of iPTH (<75pg/ml) appeared more frequent in older patients, while high levels of iPTH (≥600pg/ml) were mainly found in younger patients. In the diabetic group, the highest percentage of the patients (36.4%) had the iPTH level less than 75 pg/ml. The lowest serum P level (1.6±0.6mmol/L) was found in aged group. Serum Ca levels did not show significant differences among groups (Table 2).

**Table 2:** Patient characteristics by baseline iPTH category (n = 8530).

Patient characteristics	<75 pg/ml	75-150pg/ml	150-300pg/ml	300-600pg/ml	≥600pg/ml	P value
Age(years)	59.5±15.2	57.9±14.4	56.3±14.6	53.6±14.4	48.6±14.5	<0.01
gender						<0.05
Female	688(49%)	748(49%)	1015(45%)	930(46%)	610(46%)	
Male	715(51%)	777(51%)	1240(55%)	1091(54%)	716(54%)	
Primary cause of ESRD						<0.001
Glomerulonephritis	490(34.9%)	551(36.1%)	828(36.7%)	840(41. 6%)	658(49.6%)	
Hypertension	233(16.6%)	284(18.6%)	442(19.6%)	380(18.8%)	279(21.0%)	
Diabetic nephropathy	510(36.4%)	515(33.8%)	720(31.9%)	559(27.7%)	270(17.4%)	
Polycystic kidney disease	54(3.9%)	60(3.9%)	117(5.2%)	119(5.9%)	86(6.5%)	
Unknown	116(8.3%)	115(7.6%)	148(6.6%)	123(6.1%)	73(5.5%)	
Serum calcium (mmol/L)	2.26±0.29	2.23±0.24	2.20±0.26	2.21±0.27	2.27±0.28	<0.001
Serum phosphorus (mmol/L)	1.60±0.56	1.66±0.56	1.73±0.55	1.85±0.56	2.04±0.59	<0.001
N (%)	1403 (16. 5%)	1525 (17.9%)	2255 (26.4%)	2021 (23.7%)	1326 (15.5%)	

Mean ± standard deviation are described if the variable is normally distributed.

**Fig 1:**
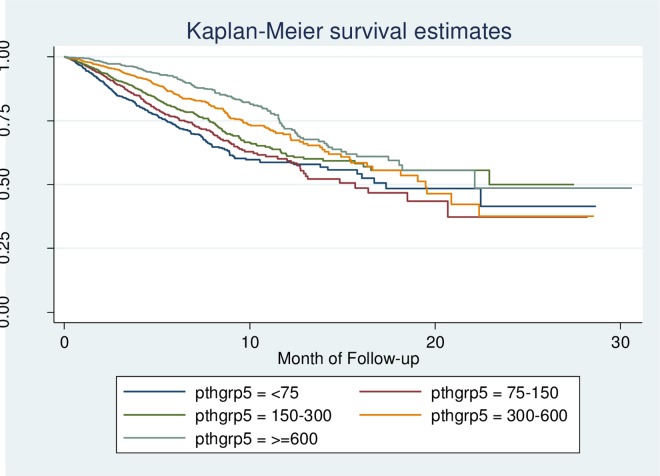
Kaplan-Meier curves for the five groups categorized by different iPTH level.

As shown in Fig 1, there were no significant differences in survival estimates among the five groups. However, Kaplan-Meier curve is a non-parametric analysis and thus it did not rule out the presence of confounding factors. Therefore, Cox regression model was adopted to analyze the risk factors of all-cause mortality.

### Risk factors of all-cause mortality

All selected parameters used for analysis except gender and serum P at baseline, were related to all-cause mortality after adjustment (Table 3).

**Table 3:** Results of baseline multivariate Cox regression and competing risk regression analysis for all-cause mortality in Beijing maintenance hemodialysis patients (n = 8530).

	Cox regression analysis		Competing risk regression analysis	
Patient characteristics	HR (95% CI)	P-value	HR(95% CI)	P-value
iPTH (pg/ml)				
<75	1.36(1.16–1.60)	<0.001	1.34(1.14–1.58)	<0.001
75–150	1.22(1.03–1.44)	0.02	1.19(1.01–1.41)	0.04
150–300	1.00		1.00	
300–600	0.86(0.73–1.01)	0.07	0.87(0.74–1.03)	0.10
≥600	0.84(0.69–1.03)	0.09	0.87(0.72–1.05)	0.14
Serum calcium (mmol/L)				
<2.1	1.54(1.37–1.74)	<0.001	1.51(1.34–1.70)	<0.001
2.1–2.5	1.00		1.00	
2.5–2.75	0.64(0.52–0.80)	<0.001	0.65(0.53–0.81)	<0.001
≥2.75	0.63(0.44–0.91)	0.01	0.65(0.46–0.92)	0.02
Serum phosphorous (mmol/L)				
<1.13	1.16(1.37–1.74)	0.10	1.18(0.98–1.41)	0.08
1.13–1.45	1.00		1.00	
1.45–1.78	0.86(0.73–1.03)	0.10	0.86(0.72–1.02)	0.08
≥1.78	0.89(0.76–1.04)	0.15	0.69(0.76–1.03)	0.13
Age (years)				
<50	1.00		1.00	
50–70	2.58(2.19–3.03)	<0.001	2.60(2.19–3.07)	<0.001
>70	6.69(5.58–8.02)	<0.001	6.68(5.52–8.10)	<0.001
Gender	1.08(0.96–1.21)	0.19	1.07(0.96–1.20)	0.24
Primary cause of ESRD				
Hypertension	1.54(1.30–1.82)	<0.001	1.51(1.28–1.78)	<0.001
Diabetic nephropathy	2.81(2.42–3.25)	<0.001	2.69(2.32–3.13)	<0.001
Glomerulonephritis	1.00		1.00	
polycystic kidney disease	0.82(0.59–1.15)	0.25	0.82(0.60–1.13)	0.23
Unknown	1.51(1.20–1.90)	<0.001	1.50(1.17–1.92)	0.001

Adjusted for age, gender, primary causes of end-stage renal disease and laboratory parameters (serum calcium, phosphorus and parathyroid hormone)

**Fig 2:**
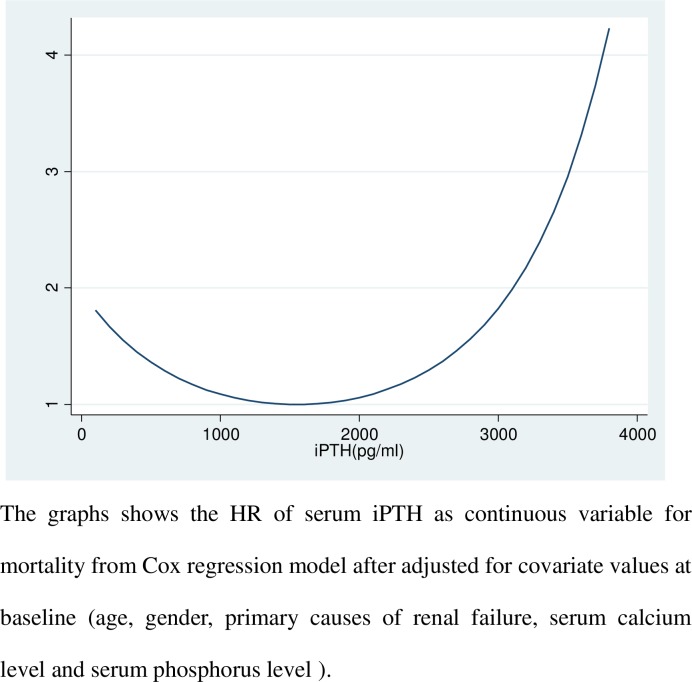
Hazard Ratio of serum iPTH as continuous variable in the Cox regression model.

Results from the Cox regression model analysis indicated that patients in the group with iPTH level <75pg/ml had greater risk of mortality [HR 1.36; 95% confidence interval (CI) 1.16–1.60, p<0.001] than those patients in the group with iPTH level within target range recommend by KDOQI (150–300 pg/ml, Table 3). The group with iPTH level ranging from 75 to 150 pg/ml also had greater risk of death compared with the target range group [HR 1.22; 95% CI 1.03–1.44, p = 0.02]. However, groups with high iPTH level (ranging from 300 to 600 pg/ml or more than 600 pg/ml) did not show an increased risk of death (p>0.05, Table 3). Similar results were observed in the competing risk regression analysis (Table 3). When iPTH was analyzed as continuous variable, U-shaped relationship was found in this model (Fig 2). As shown in Fig 2, the risk of mortality increased when iPTH was more than 2800 pg/ml and less than 80pg/ml. Hazard Ratio (HR) of serum iPTH as continuous variable was evaluated by the Cox regression model. The graphs showed the HR of serum iPTH as continuous variable for mortality after adjusted for covariate values at the baseline (age, gender, primary causes of renal failure, serum Ca and serum P level).

Results were similar using both Cox regression analysis and competing risk model analysis for serum Ca. The group with low levels of serum Ca (<2.1mmol/L) was associated with a higher risk of mortality than the group with target range serum Ca (2.1–2.5mmol/L) [HR 1.54; 95% CI 1.37–1.74, p<0.001 and HR 1.51, 95% CI 1.34–1.70, p<0.001, respectively]. Meanwhile, groups with higher serum Ca level group (2.5–2.75mmol/L) had a lower risk of death than the target range group [HR 0.64; 95% CI 0.52–0.80, p<0.001 and HR 0.65; 95% CI 0.53–0.81, p<0.001 respectively]. Patients with baseline serum Ca levels equal to or over 2.75 mmol/L also had a lower risk of mortality than those within the target range [HR 0.63, 95% CI 0.44–0.91, p = 0.01 and HR 0.65; 95% CI 0.46–0.92, p = 0.02 respectively].

As for serum P, either lower or higher levels were not related to the risk of mortality, when compared to target range serum P group (p>0.05). The results were also confirmed by both Cox regression model and competing risk model analyses.

### Age, gender, primary cause of ESRD and the risk of mortality

Patients over 70 years old experienced nearly 7-fold increase in risk of mortality, compared with those who were younger than 50 years of age. The risk reduced to nearly 3-folds when the patients were between 50 to 70 years of age (Table 3). No significant differences were found regarding the risk of death between males and females.

Data from Cox regression model analysis with chronic glomerulonephritis as reference 1, revealed that the causes of ESRD including primary hypertension (HR 1.54; 95%CI 1.30–1.82, p<0.001), diabetic nephropathy (HR2.81; 95%CI 2.42–3.25, p<0.001), and other unknown factors (HR1.51; 95%CI 1.20–1.90, p<0.001) could increase the risk of mortality. Competing risk regression model analysis provided similar results.

## Discussion

To our knowledge, this was the largest cohort study to evaluate CKD-MBD markers and their association with the outcomes carried out in the Chinese prevalent hemodialysis population. Competing risk regression model was applied in this study. KDOQI guidelines were generally adopted in the clinical practice in China prior to 2013 and so were used in this study as a reference for analysis. Considering the target ranges recommended by KDOQI guidelines as reference, we noticed 19% and 34% increase in HR for mortality in patients with iPTH levels ranging from 75 to 150 pg/ml or less than 75 pg/ml respectively. A 51% increase in HR was also demonstrated in patients with serum Ca level under 2.1 mmol/L. However, a 35% reduction in HR was identified in patients with serum Ca level over 2.5 mmol/L at baseline. The mortality rate was 14.6%, similar to that (13.9%) reported by Cheng et al in 2013[8]. Ageing (≥50 yrs),diabetes and hypertension which were presented as primary causes of ESRD, all remained as strong predictors of mortality.

The categorical variables model analysis showed that lower iPTH levels (<150 pg/ml) but not higher iPTH levels were associated with higher risk of all-cause mortality in our study which is in disagreement with findings from previous studies [1–5, 9]. Two studies from the United States [1, 9] reported that higher iPTH levels (≥600 pg/ml) were associated with high mortality risk. Tentori et al [2] also reported in DOPPS—a worldwide baseline data analysis—that the greatest risk of mortality was found for iPTH levels greater than 600 pg/ml. However, in the HEMO study, no correlation was found between iPTH levels and the mortality risk [3]. U-shaped correlation between the iPTH level and all-cause mortality in hemodialysis patients were reported in two historical cohort studies in Latin America and Europe [4, 5]. However, two other prospective studies supported the results in the present study. Results from the first one, evaluating 8,377 hemodialysis patients in France, showed that only low iPTH (<130 pg/ml) was predictive to mortality [10]. The other one, a 14-year prospective observational study evaluating 345 hemodialysis patients and 277 peritoneal dialysis patients, indicated that lower levels of iPTH (65 to 199 pg/ml and lower than 65 pg/ml) in uremic patients was associated with increased mortality [11].

Nevertheless, results from Cox regression analysis with continuous variable for serum iPTH indicated the existence of an U-shaped relationship between serum iPTH level and mortality. Both the CORES study and European study [4, 5] supported the similar results in spite of the existing different cut-off values.

Possible reasons for this discrepancy between our study and other studies were as follows. (1) Patient characteristics and the clinical practice pattern were different between China and other countries. (2) Patients with a higher level of iPTH (≥600pg/ml) were younger than those with a lower level of iPTH (<75 pg/ml, 48.6±14.5 vs 59.5±15.2 years old). (3) Conditions such as patients with a baseline level of iPTH ≥300pg/ml, which were either treated with medications or were submitted to parathyroidectomy during the period of follow-up program were not taken into account in this study. (4) In our study, differences observed when results from categorical analysis and continuous variable analysis were compared are possibly due to: information was lost in the categorical analysis, and there were few patients with extremely high serum iPTH (>2000 pg/ml).

In the present study iPTH levels between 1000 and 2000 pg/ml were related to the lowest HR for all-cause mortality. The range is quite outside of the recommended iPTH levels by both KDOQI and KDIGO guidelines. One of limitations in the present study is the lack of information for cases of parathyroidectomy in our subjects. A recent study from Taiwan demonstrated that parathyroidectomy reduces the death risk in hemodialysis patients [12]. A recent research showed that successful parathyroidectomy might reduce the risk for all-cause and cardiovascular mortality in hemodialysis patients with severe, uncontrolled SHPT [13].

Even considering that patients with iPTH under 75 pg/ml were the oldest among the five groups, lower level of iPTH was still associated with all-cause mortality after the adjustment of age. It is well known that cardiovascular disease is the most common condition causing death in patients on dialysis [14], and vascular calcification is the major mechanism of cardiovascular disease in these patients [15, 16]. Several studies have proved that hyperparathyroidism can accelerate vascular calcification at the stage of ESRD [17, 18]. Moreover, low iPTH is also an independent risk factor that predicts the vascular calcification [19].

Previous results are inconsistent regarding to the correlation between serum Ca concentration and relative risk of death. Some studies suggested that high serum Ca increased the risk of mortality[1, 3]. While several studies, based on time-dependent analysis, indicated that both high and low serum Ca levels were associated with high risk of mortality [2, 5, 9]. Results from our baseline analysis supported that low serum Ca level (<2.1 mmol/L) was a predictive factor of high risk on the all-cause mortality and high serum Ca level (≥2.5mmol/L) at baseline decreased the risk of mortality. The reason is unknown. A possible explanation is that patients with higher serum Ca levels at baseline were patients with SHPT and underwent therapy. Further studies are needed to validate the evidence in the future.

Both low and high levels of serum P had been reported as risk factors for mortality in hemodialysis patients[1–3, 5]. However, in a prospective observational study conducted among French hemodialysis patients, serum P was not proved to be of predictive value on mortality by adjusted Cox analyses[10]. Our study also showed that the baseline serum P level was not related to the risk of mortality.

There are limitations in this study. First of all, this is a retrospective observational study rather than a randomized controlled study. Secondly, as stated in a previous study [8], the Beijing hemodialysis dataset only referred to city but not national. Some primary causes of ESRD were uncertain. Therefore, more detailed analysis between primary cause of kidney disease and mortality were not available. Thirdly, the potential protective effects of medications and surgery such as Vitamin D supplement and parathyroidectomy were lack of being recorded.

Strengths of this study would include large sample size, longer-term of follow- up programs, and a farely reliable database set in Beijing. Another key point in our study is the successful adoption of the competing risk regression model[20]. Since some of the patients in this study had undergone renal transplantation or being transferred to peritoneal dialysis, these events should be considered as competing risk events. In our study, the results from both Cox regression analysis and Competing risk regression analysis appeared almost the same.

In conclusion, the current study demonstrated that patients with lower levels of serum iPTH or Ca were at high risk of mortality compared with those with target range recommended by KDOQI. Our findings suggested that aggressive management of patients with lower iPTH or Ca level at baseline might be of importance.

## Supporting Information

S1 Dataset The relevant original dataset of this study (original data.xls). (XLS)
